# Explanations for female excess psychosomatic symptoms in adolescence: evidence from a school-based cohort in the West of Scotland

**DOI:** 10.1186/1471-2458-7-298

**Published:** 2007-10-22

**Authors:** Helen N Sweeting, Patrick B West, Geoff J Der

**Affiliations:** 1 MRC Social and Public Health Sciences Unit, 4, Lilybank Gardens, Glasgow, G12 8RZ, UK

## Abstract

**Background:**

By mid adolescence there is an excess in female physical and/or psychosomatic, as well as psychological morbidity. This paper examines the contribution of a range of factors (self-esteem, body image, gender-role orientation, body mass index, smoking and physical activity) to explaining the female excess in three psychosomatic symptoms (headaches, stomach ache/sickness, and dizziness) and depressive mood at age 15.

**Methods:**

A cohort of 2,196 school pupils (analyses restricted to 2,005 with complete data) surveyed at age 15. All measures were obtained via self-completion questionnaires, apart from body mass index, derived from measured height and weight. Analyses examined (a) sex differences in each potential explanatory factor; (b) their associations with the health measures; (c) the effect of adjustment for these factors on sex differences in the health measures; and (d) the existence of interactive effects between sex and the explanatory factors on the health measures

**Results:**

Each potential explanatory factor was significantly differentiated by sex. Self-esteem, body image (represented by weight-related worries), smoking and physical activity were related to the health measures. These factors accounted for one third of the female excess in headaches and stomach problems, half the excess in dizziness and almost all that in respect of depressive mood. Self-esteem and body image were the factors most consistently related to health, and adjustment for these resulted in the largest reductions in the odds of a female excess in both the psychosomatic symptoms and depressive mood.

**Conclusion:**

Adjustment for a range of potential psychosocial and behavioural factors largely explains (statistically) excess female depressive mood. These factors also partially explain the female excess in certain psychosomatic symptoms.

## Background

The emergence of higher rates of psychological distress among females in early-mid adolescence is well documented [1-3], one study showing a consistent pattern in the onset of excess depression among females at age 14 across three Western countries, irrespective of how measured [4]. Other studies have found a similar pattern for chronic illness, self-reported health, physical and/or psychosomatic symptoms [5-8]. Thus, in previous analyses, based on the dataset employed in this paper, we showed that generally high levels of self-reported morbidity tended to increase between ages 11 and 15, these increases being greater for females. The result was that by age 15, there was a significant female excess in not only depressive mood and the (past month) 'malaise' symptoms of nervousness, irritability, sadness and sleeping problems, but also in limiting illness, poor self-rated health and the more 'physical' symptoms of headache, stomach problems, feeling dizzy or faint, and cold or flu (but not aching joints, skin problems or asthma/wheeze) [7].

With the exception of colds or flu where the sex difference, though significant, was small, the 'physical' symptoms which showed a female excess (headaches, stomach problems and dizziness) might be better described as psychosomatic. By psychosomatic, we mean physical symptoms less likely to reflect organic disease, and more likely to comprise a substantial psychological component [9,10]. In so doing, we acknowledge that the physical/psychological distinction is necessarily somewhat arbitrary [11]. In contrast, significant sex differences were not apparent among the more 'physical' symptoms available within our dataset (aches, skin problems and asthma/wheeze).

Explanations for the female excess in physical and/or psychosomatic morbidity evident by mid adolescence have received little attention [12]. However, sex differences in adult health have been explained in terms of different roles, stresses, expectations, reporting behaviours, lifestyles and health practices, as well as biology [13]. Similar explanations have been advanced in the case of depression among adolescent females [2,14].

In a key paper on mechanisms underlying sex differences, in this case in psychopathology, Rutter and colleagues argued that in order to identify factors underlying sex differences in any disorder, a number of criteria must be met. These are either that there are different exposures (different levels/rates) associated with similar levels of risk/protection within each sex (which we term the exposure hypothesis), or similar exposures associated with different levels of risk/protection (termed here the susceptibility hypothesis). Further, the sex difference in the disorder must be eliminated or reduced after adjustment for the factors [3]. These two hypotheses, exposure and susceptibility, can also be expressed in terms of mediators and moderators more familiar within psychology literature. Mediators (intermediate variables) explain how or why another variable affects an outcome. Applying this terminology, the identification of a potential explanatory factor as a mediator of a sex difference in a health outcome requires that (a) it is related to sex (i.e. levels of *exposure *are differentiated by sex), (b) it is related to the health outcome, and (c) exactly as identified by Rutter and colleagues, the association between sex and the health outcome is eliminated or reduced once the factor is controlled for. Moderators (effect modifiers) affect the relationship between a variable and an outcome. If sex acts as a moderator (i.e. one sex is more *susceptible *to a particular explanatory factor) then there will be an interactive effect between sex and that factor on the health outcome [15-17].

This paper aims to further our understanding of the female excess in psychosomatic symptoms in mid adolescence, by examining a range of potential explanatory factors. Some (self-image, gender-role orientation, body shape) have previously been put forward as possible explanations for female excess psychological distress [2,14]; others (health-related behaviours) have not. These factors are discussed below.

A number of psychosocial mechanisms may potentially explain female excess psychosomatic symptoms. First of these is ***self-image***, represented by ***self-esteem ***and ***body image***, both of which have been associated with depression [18,19], indeed it has been suggested that positive self-esteem and depression form opposite ends of a single continuum [20]. Overall self-esteem has been associated with symptoms including sleep problems, tiredness, lethargy and sore muscles among adolescents [5] and self-rated physical health among students [21], while adults with greater bodily self-esteem have been found to score higher on a scale of 'wellness' [22]. Self-esteem is differentiated by sex [23,24], being lower among females, and sex differences in body image dissatisfaction (e.g. weight-related worries) increase during early-mid adolescence [25,26]. We might thus expect the lower self-esteem and poorer body image of adolescent females to contribute to their greater psychosomatic, in addition to psychological morbidity.

The second psychosocial mechanism, ***gender-role orientation (GRO)***, refers to an individual's 'masculinity' (characterised by traits such as aggressiveness, dominance and independence) or 'femininity' (traits such as warmth, sensitivity and nurturing). Studies of both children and adults generally show masculinity and/or androgyny (high masculinity *and *femininity) to be associated with better physical and psychological well-being. Since males tend to have higher masculinity, and females higher femininity, the implication is that gender-role may (partly) account for excess psychosomatic symptoms among females [27-32].

Another potential explanatory factor for female excess psychosomatic symptoms is ***body shape***. During early-mid adolescence, females experience a gain in fat associated with puberty, while maximum body mass index (BMI) occurs later in males [33]. The vast number of studies in respect of the psychological consequences of high levels of BMI in childhood and adolescence have produced mixed findings [34-36]. While some find overweight or obesity to be associated with reduced self-esteem or increased depression, at least among females [37,38], others, including an analysis of the present sample, find no evidence of such a relationship [39]. There is also some evidence to suggest reduced well-being among underweight adolescent males, attributable to Western society's ideal male muscular physique [40]. In respect of physical health, obesity is associated with cardiovascular, metabolic and orthopaedic problems, as well as self-assessed fitness and health [36,41]. It is therefore possible that sex differences in BMI, or in any relationship which it may have with psychosomatic symptoms, could help to account for the female excess in those symptoms.

Health-related behaviours comprise a further potential explanatory factor for female excess psychosomatic symptoms. ***Smoking ***is associated with poorer adolescent self-rated physical health [21] and psychological well-being [42-44]. Further, although perhaps not so clearly as in adults [45]*** physical activity ***in young people is associated with weight loss [46] and better psychological well-being, as indicated by factors such as self-esteem and mood [47-49]. The pattern of sex differences in both behaviours suggests they might also contribute to excess female psychosomatic symptoms as well as psychological morbidity; in Scotland, by mid-adolescence, rates of smoking are higher in females and physical activity in males [50].

Our analyses examine whether these factors explain a female excess in three psychosomatic symptoms at age 15. These include headache and stomach problems (being the two most 'gendered' [8]), and dizziness (for which a female excess emerged in this cohort between 11 and 15 [7]). For comparison, we also include depressive mood. Consistent with the concept that psychosomatic symptoms comprise a substantial psychological component, relationships between depressive mood and each symptom were all significant, the strongest associations occurring in respect of dizziness. Thus, rates of headache among those defined as suffering depressive mood compared with those who were not (see Methods) were 78% and 62% respectively in males, 90% and 78% in females; rates of stomach problems were 75% and 50% (males), 89% and 75% (females); rates of dizziness were 54% and 24% (males), 34% and 54% (females).

An alternative to analysing individual symptoms would have been to create a symptom score, or a variable representing 'high' psychosomatic symptoms. We have not adopted this method for two reasons. Firstly, just as sex differences in health vary according to the measure in question, so too might we expect the range of factors which determine those differences to vary, and this is something which we wish to explore. Secondly, it is possible that factors associated with a general propensity to report high numbers of symptoms differ from those associated with reporting any one individual symptom.

In line with suggestions for identification of factors underlying sex differences in health [3] and recommendations for the investigation of mediating or moderating effects [15-17], we suggest four hypotheses. The first three relate to what we term 'exposure', and the last to 'susceptibility' explanations:

1. There will be sex differences in each of the potential explanatory factors; that is, each explanatory factor will be related to sex.

2. These explanatory factors will be related to our measures of health, although these relationships are likely to differ according to the health measure in question. Thus, while we might, for example, expect physical activity to be associated with depressive mood, there is no a priori reason to think it would be related to headache. However, for completeness, we begin by examining the relationship that each explanatory factor has with each health measure, so contributing to the rather scarce [21] literature on predictors of adolescent self-rated physical health.

3. These factors will contribute to the (statistical) explanation of the sex differences in our measures of health; that is, the sex differences will be reduced or eliminated once the factor is controlled for.

4. Relationships between the potential explanatory factors and the health measures may also differ for males and females; that is, there may be an interactive effect between sex and the explanatory factors on the health measures.

## Methods

### Sample

Data are from the *West of Scotland 11 to 16 Study*, a longitudinal study of health and lifestyles in a single school year cohort [51]. The study received approval from Glasgow University's Ethics Committee, participating Education Authorities and schools, and consent was obtained from the parents of all participants.

Participants were recruited in their final year of primary school (age 11) and followed through the transition to secondary school until the end of statutory education (age 15). The sampling scheme involved a number of steps to ensure a representative sample at both the primary and secondary school stages, taking into account the fact that increasing parental choice has diminished the traditional links between local associated primary and secondary schools. Secondaries were randomly selected within strata based on geographical location, religious status (Catholic/non-denominational) and deprivation; primaries on the basis of the proportion of pupils transferring to the selected secondaries, and finally classes of children within primaries according to the first letter of the class teacher's name [52].

At age 11, 2,586 children (93% of the issued sample) were surveyed. The age 15 follow-up, conducted in 1999, included 2,196 (85% of baseline, and 79% of the issued sample). The baseline sample was representative of the population in respect of sex and social class, thereafter differential attrition (e.g. persistent school truants) made it less so. Probabilistic weights were derived to correct for this [53], but because these made only very minor differences to the results, those based on unweighted data are presented here.

The school-based survey included self-completion questionnaires administered in exam-type conditions. Nurses helped with completion if necessary, conducted a short interview and recorded physical measurements.

### Measures

All measures of relevance to this paper were obtained at age 15.

### Health

Each health measure was a simple binary outcome (see Table 1). The questionnaire contained a list of 11 symptoms, from which respondents indicated any they had suffered in the previous month. These included '***headache'***, '***stomach ache or sickness' ***and ***'felt dizzy or faint'***. The questionnaire included a brief index of ***depressive mood ***[54]. This asked how often each of six items (e.g. too tired to do things; unhappy, sad or depressed) had been experienced in the last month (most of the time, sometimes, never), the index of depressive mood being based on the average score multiplied by 10. 'Caseness' was identified via a cut-off suggested by the authors (21.8 – the mean obtained in their own clinical adolescent sample).

**Table 1:** Sex differences in each measure of poor health and each potential explanatory factor.

	**males**	**females**		***(sig)***
(N)	(1023)	(982)		
**HEALTH MEASURES**				
headache	62.9%	80.2%	χ^2 ^= 73.7	*(.000)*
stomach, sick	52.1%	77.8%	χ^2 ^= 144.5	*(.000)*
dizzy, faint	25.9%	37.1%	χ^2 ^= 29.3	*(.000)*
depressive mood	7.0%	17.2%	χ^2 ^= 49.0	*(.000)*
**POTENTIAL EXPLANATORY FACTORS**				
self-esteem (z-score)	.30	-.32	F = 214.3	*(.000)*
worried about putting on weight	22.9%	70.8%	χ^2 ^= 462.3	*(.000)*
masculinity (z-score)	.20	-.21	F = 90.3	*(.000)*
femininity (z-score)	-.17	.17	F = 59.2	*(.000)*
BMI (z-score)	-.13	.14	F = 37.8	*(.000)*
current smoking	20.0%	27.7%	χ^2 ^= 16.2	*(.000)*
physical activity (z-score)	.23	-.24	F = 122.2	*(.000)*

### Potential explanatory factors

***Self-esteem ***– respondents completed a scale, based on Rosenberg's [55], which included 10 items such as 'I am pretty sure of myself', with a 4-point response (strongly agree – strongly disagree). Scores among this sample ranged 5–30 (mean = 19.9, SD = 4.1).

***Body image (weight-related worries) ***– respondents were asked 'Are you worried about putting on weight?', with dichotomous yes/no response categories.

***Gender-role orientation (GRO) ***– time constraints, plus critical comments from children in pre-pilot work, resulted in a decision not to include a full children's or young people's [56,57] gender-role scale. However, six items representing those with particularly high weightings on factors corresponding to femininity and masculinity on the adult Bem Sex Role Inventory [27,58] and versions for children and young people [56,57] were included. Three represented 'femininity' ('I am kind', 'I care about others' and 'I am gentle') and three masculinity ('I stand up for myself', 'I am a leader' and 'I am tough'). These were measured with a 4-point response (very untrue – very true) and summed to produce two scales each with a range 3–12 (masculinity – mean = 7.6, SD = 1.4; femininity mean = 9.4, SD = 1.1). The Chronbach's alphas 54 for masculinity and .69 for femininity) for these scales are below the conventionally desirable level of .70 [59].

***BMI ***– nurses measured height and weight, used in the calculation of BMI (kg/m^2^) (range = 13.9–44.9, mean = 21.2, SD = 3.5).

***Smoking ***– respondents were categorised as current smoker (occasional or regular) or non-smoker (never or ex).

***Physical activity ***– respondents were asked how often (using a 5-point scale, range never – every day) they took part in a list of 10 activities outside school (e.g. 'football', 'gymnastics, ballet or dancing') and up to three additional (unlisted) activities. The physical activity score is the sum of the items (range = 13–46, mean = 23.1, SD = 5.2).

### Analyses

• Hypothesis 1: Chi-square or the F-test were used to determine whether there were sex differences in each of the potential explanatory factors.

• Hypothesis 2: Logistic regression analyses were conducted to examine associations between the explanatory factors and the health measures.

• Hypothesis 3: Logistic regression analyses were conducted to examine whether inclusion of the explanatory factors (singly and in combination) in the model reduced or eliminated sex differences in our measures of health.

• Hypothesis 4: Further sets of logistic regression analyses were conducted, entering all explanatory factors in combination, plus the interaction between sex and each factor singly (i.e. all factors plus sex by self-esteem; all factors plus sex by weight-related worries; etc) in respect of each health measure, to determine whether relationships between the explanatory factors and the health measures differed according to sex.

Because of the large number of separate analyses, we adopted a stricter level of significance than is usual, only considering results with p < .01 as significant. Although the sample is clustered by school, the intraclass correlations are low (0.01 for sex and 0.002 to 0.01 for the outcomes), and design effects are, therefore, unlikely to affect the results. All analyses were restricted to those with complete data in respect of each explanatory factor (1,023 males, 982 females). The self-esteem, femininity, masculinity and physical activity scores, together with BMI, were transformed to z-scores (zero mean and unit standard deviation), to enable comparison of the strength of their associations with the health measures.

## Results

### Sex differences in each of the potential explanatory factors

Table 1 shows the female excess in headache, stomach problems, dizziness and depressive mood, and sex differences in each potential explanatory factor. Females had lower self-esteem, were more likely to worry about putting on weight, and had lower masculinity but higher femininity scores. Further, BMIs were higher, smoking more common, and physical activity less frequent for females.

### Associations between the health measures and each potential explanatory factor

Table 2 shows the association between the health measures and each potential explanatory factor. The strongest, most consistent relationships occurred in respect of self-esteem and worries about putting on weight. One standard deviation increase in self-esteem reduced the odds of headache, stomach problems and dizziness by approximately a quarter, and of depressive mood by around two-thirds. Rates of each symptom were doubled, and those of depressive mood trebled among those worried about putting on weight. The odds of each health measure, particularly depressive mood and headache were also higher amongst current smokers, while more frequent physical activity was associated with lower levels of each measure except dizziness. Although the odds of depressive mood were raised among those with higher femininity scores, and of headache among those with greater BMIs, neither of these relationships was significant at the .01 level. Additional analyses (not shown), examining associations with obesity, defined according to the UK90 cut-offs for age and sex [60], found no relationship with any of the health measures. Finally, there were no associations between masculinity and any health measure.

**Table 2:** Univariate associations, expressed as odds ratios (and 99% CIs) between each measure of poor health and each potential explanatory factor.

	**headache**	**stomach, sick**	**dizzy, faint**	**depressive mood**
(N)	(2003)	(1999)	(1999)	(2005)
**self-esteem score **#	0.76 (0.67–0.87)	0.69 (0.60–0.78)	0.72 (0.63–0.82)	0.37 (0.30–0.45)
**worried about putting on weight **~	1.88 (1.44–2.44)	2.23 (1.74–2.86)	1.78 (1.39–2.29)	3.17 (2.16–4.66)
**masculinity score **#	0.96 (0.85–1.09)	0.95 (0.84–1.07)	0.99 (0.87–1.12)	0.89 (0.75–1.07)
**femininity score **#	1.08 (0.95–1.23)	1.03 (0.92–1.17)	1.02 (0.90–1.16)	1.14 (0.96–1.36)
**BMI **#	1.11 (0.97–1.27)	1.05 (0.93–1.19)	1.01 (0.89–1.15)	1.01 (0.85–1.21)
**current smoking **~	1.75 (1.26–2.42)	1.62 (1.21–2.19)	1.40 (1.05–1.85)	1.87 (1.28–2.72)
**physical activity score **#	0.82 (0.72–0.93)	0.80 (0.70–0.90)	0.93 (0.82–1.05)	0.73 (0.60–0.88)

# = O.R. in respect of SD increase.

~ = O.R. in respect of worried (Vs not worried)/current smoker (Vs non-smoker)

The combination of lower self-esteem, higher rates of weight worries and smoking, and lower levels of physical activity, together with the associations between these variables and health, suggest their potential as explanations for a female excess in certain morbidity measures. In contrast, BMI, masculinity and femininity had little or no explanatory power.

### Were sex differences in the health measures reduced or eliminated after controlling for the potential explanatory factors?

Table 3 shows odds of a female excess of each morbidity measure. It enables a comparison of the unadjusted odds with those after adjustment for each of the factors that had emerged as significantly associated with that particular measure, both separately and in combination. The final row shows the odds of a female excess after adjustment for all relevant factors. Table 3 also shows the percentage of the female excess explained (in statistical terms) by each factor separately and in combination.

**Table 3:** Odds ratios (and 99% CIs) of a female excess of each measure of poor health – unadjusted, and after controlling for all factors separately and in combination.

	**headache**	**stomach, sick**	**dizzy, faint**	**depressive mood**
*(N)*	*(2003)*	*(1999)*	*(1999)*	*(2005)*
**UNADJUSTED**				
OR (95% CIs)	2.39 (1.84–3.12)	3.22 (2.50–4.16)	1.69 (1.31–2.17)	2.75 (1.87–4.03)
**ADJUSTED FOR ...**				
				
**self-esteem score**				
OR (95% CIs)	2.19 (1.66–2.89)	2.84 (2.18–3.71)	1.43 (1.10–1.86)	1.64 (1.09–2.47)
*% female excess explained*	*14%*	*17%*	*38%*	*63%*
				
**worried about putting on weight**				
OR (95% CIs)	2.10 (1.55–2.83)	2.76 (2.07–3.67)	1.38 (1.04–1.84)	1.83 (1.19–2.81)
*% female excess explained*	*21%*	*21%*	*45%*	*53%*
				
**current smoking**				
OR (95% CIs)	2.33 (1.78–3.04)	3.15 (2.44–4.07)	1.65 (1.29–2.13)	2.64 (1.80–3.88)
*% female excess explained*	*4%*	*3%*	*6%*	*6%*
				
**physical activity score**				
OR (95% CIs)	2.28 (1.74–2.99)	3.07 (2.36–3.99)		2.49 (1.68–3.69)
*% female excess explained*	*8%*	*7%*		*15%*
				
**ALL ABOVE VARIABLES**				
OR (95% CIs)	1.88 (1.38–2.57)	2.44 (1.81–3.27)	1.23 (0.92–1.65)	1.20 (0.75–1.92)
*% female excess explained*	37%	35%	67%	*89%*

Only two factors, self-esteem and weight worries, consistently reduced the female odds. These reductions were most in respect of depressive mood, followed by dizziness. Much smaller reductions in the female odds were seen after adjusting for smoking and physical activity, the latter explaining more of the female excess in depressive mood than in either headache or stomach problems. Adjustment for all relevant factors accounted for around one-third the female excess in headache and stomach problems, two-thirds the female excess in dizziness and almost all that in depressive mood.

### Did relationships between the explanatory factors and the health measures differ according to sex?

In respect of the susceptibility hypothesis, to examine whether sex acted as a moderator, affecting the relationship between the explanatory factors and the health measures, the significance of each sex by explanatory factor interaction on each health measure was examined (results not shown). Only one of these was significant; sex by masculinity on depressive mood (p = .001). To investigate this further, the masculinity (z-) score was split at the 50^th ^percentile; 59.0% of males and 39.5% of females falling into the 'high' category. Among those with 'low' masculinity, there was no significant female excess in depressive mood, the unadjusted OR (and 99% CIs) being 1.63 (0.96–2.76), reducing to 0.75 (0.38–1.47) after adjustment for self-esteem, weight worries, smoking and physical activity. Equivalent ORs among those with 'high' masculinity were 4.78 (2.73–8.37) reducing to 2.14 (1.10–4.17) after adjustment. Higher masculinity was therefore associated with significantly increased rates of depressive mood in females.

## Discussion

This paper has examined the role of various potential explanations for excess female self-report psychosomatic symptoms and depressive mood in mid adolescence. We hypothesised that there would be sex differences in each of the potential explanatory factors ('exposure'), that these factors would be related to our measures of health, and that they would contribute to the (statistical) explanation of the sex differences in our measures of health. Further, we suggested that relationships between these factors and our measures of health might differ for males compared with females ('susceptibility').

The first hypothesis was substantiated; there were significant sex differences in exposure to each potential explanatory factor. In relation to our second hypothesis, four of the factors (self-esteem, body image as represented by weight-related worries, smoking and physical activity) were related to the health measures, but three (masculinity, femininity and BMI) were not. In respect of our third hypothesis, taken together, these factors could account for one third of the female excess in headaches and stomach problems, two-thirds the excess in dizziness and almost all that in respect of depressive mood. Finally, (fourth hypothesis) there was a significant sex by masculinity interaction on depressive mood, demonstrating that this relationship differed for males compared with females. The results suggest that it is largely the exposure hypothesis which is operating in respect of the health measures included in our analyses.

We suggested that the relationships between the explanatory factors and health, and the degree to which they explained female excess psychosomatic morbidity, were likely to differ according to the measure in question. The factors most consistently related to our health measures were self-esteem, followed by weight-related worries (representing body image), and it was generally only these which made any impact on sex differences in health. Of the health measures included in our analyses, the greatest reductions in the female excess occurred in respect of depressive mood. However, some of the female excess in headache, stomach problems and, more particularly, dizziness, could be explained by self-esteem and weight worries. This underlines the arbitrariness of the distinction between 'physical' versus 'psychological' health or symptomatology [10,11].

Of the other potential explanatory factors, neither femininity nor BMI was significantly associated with any health measure. Although the health-related behaviours were associated with each health measure, they made only small (physical activity) or very small (smoking) contributions to the female excess in either psychosomatic symptoms or depressive mood.

Overall, our results suggest little support for the susceptibility hypothesis. The only exception occurred in respect of masculinity, which was positively associated with depressed mood in females only. However, since levels of masculinity were higher in males, this factor cannot explain female excess depressive mood in this sample. The finding of a positive association between masculinity and depressive mood in females is consistent with earlier suggestions that, at least in early adolescence, masculine and feminine sex-typed adolescents may have greater self-esteem, because of their conformity with social norms [61]. However, it runs counter to the majority of studies of adolescents or young people which suggest that masculinity is associated with psychological well-being in both males and females and that differences in masculinity contribute to sex differences in depression and self-esteem [28-30,32]. Previous analyses of this sample have shown that while masculinity was associated with increased likelihood of being a bully and reduced likelihood of victimisation, loneliness and low self-esteem in both sexes, in females it was also associated with psychological distress (represented by the General Health Questionnaire – [62]). In contrast, a 'Gender Diagnosticity' [63] measure of 'maleness' based on sex differences in frequency of participation in various leisure and sports activities was associated with reduced psychological distress in both males and females [64].

It has been suggested that different findings in respect of gender-role orientation might result from the use of different measures [28]. Our gender-role orientation scales were brief and potentially lacking both reliability and validity. The majority of our potential explanatory variables, and all the health measures were self-report, and so likely to include measurement error to a greater or lesser extent. Measurement error in a mediator variable tends to result in under-estimates of its effect, which is 'not a desired outcome, because successful mediators may be overlooked' [15], p.117. Given this, it is likely that female excess morbidity could never be completely and satisfactorily explained by a study such as ours.

A further limitation of our study is the restricted range of potential explanatory factors included in our models. One of the more obvious examples of these is puberty, with several studies having found an association between pubertal status and female psychological well-being [1,65,66]. Menarche has also been associated with physical symptoms including menstrual cramps [67], headaches and migraine [68]. In contrast, male puberty brings physical and maturational changes generally regarded as positive [69]. In the current sample, puberty (assessed by voice breaking or periods starting) had been reached by 91% of males and 97% of females. The very small number of females who had not reached puberty ruled out comparisons in respect of this variable.

A final potential limitation is that of generalisability. The cohort from whom the data were gathered was representative of the population from which they were drawn. However, they were located in a particular geographic region, and surveyed at a particular point in time. The study region, the West of Scotland has a poor health record [70], raising the possibility that the meaning or significance of symptoms might differ from other geographic contexts. The data were gathered in 1999. It is possible that the growing public visibility of young women, coupled with convergence in male and female rates of substance use [71,72] may have had, and continue to have, an impact on levels of female morbidity.

Finally, we note that even where our analyses demonstrated a (partial) statistical explanation for female excess morbidity, caution is warranted in making causal inferences [16]. For example, part of any (inverse) relationship between depressive mood and physical activity may be because those who are depressed are more likely to be sedentary [73]. Similarly, the association between self-esteem and depressive mood is very firmly established, with evidence that each has an effect on the other [74,75], such that it has been suggested they may even form a continuum [20].

These results suggest that additional explanations are required for the female excess in psychosomatic symptoms evident in mid adolescence. Further, we found that it was a psychosocial factor (self-image, represented by self-esteem and body image) which contributed most to explaining sex differences in the symptoms under investigation here. This implies that studies which seek to account for a female excess in 'physical' symptoms of a less clearly psychosomatic nature would require different sets of factors. In addition to the impact of puberty detailed above, greater physical sensitivity has been put forward as an explanation for female excess physical symptoms [76], although contrary findings have also been reported [77]. There may also be cultural explanations; just as Western notions of femininity have made the display of anxiety and sadness, and talking about feelings, more acceptable for women [78], so the endorsement of certain physical symptoms may be more acceptable for females. Other authors have linked the female excess to macro-level societal characteristics, specifically male-female differences in education, income, work, political power and life expectancy [8]. From a somewhat different, and more methodological perspective, the time-frame may also be important, females being more likely to report excess symptoms retrospectively rather than currently, suggesting a 'trait-like' experience [79].

## Conclusion

Although excess female physical and/or psychosomatic as well as psychological morbidity emerges in early-mid adolescence, it has received much less attention. This paper has examined the contribution of a range of factors to explaining the female excess in three psychosomatic symptoms (headaches, stomach ache/sickness and dizziness) and, by way of contrast, depressive mood, at age 15. Only sex differences in self-image, a psychosocial factor represented here by self-esteem and weight-related worries, contributed significantly to the female excess in both the psychosomatic symptoms and depressive mood. These results suggest that additional factors are required to explain the female excess in psychosomatic morbidity evident by mid adolescence. We have previously proposed that qualitative as well as quantitative studies may be needed to most usefully explore the ways in which influences on the health of children and young people differ and change according to sex and age [7]. The results of our quantitative analyses serve only to underline this.

## Competing interests

The author(s) declares that they have no competing interests.

## Authors' contributions

HS conceived and performed this analysis and drafted the manuscript. PW and HS were both involved in the design and conduct of the *West of Scotland 11 to 16 Study*. GD provided statistical advice. All authors read and approved the final manuscript.

## Pre-publication history

The pre-publication history for this paper can be accessed here:


